# A Method for Specific Emitter Identification Based on Polarimetric Domain Feature Learning and Extraction

**DOI:** 10.3390/s26082368

**Published:** 2026-04-11

**Authors:** Zixuan Zhang, Zhiyuan Ma, Zisen Qi, Jia Liang, Hua Xu

**Affiliations:** Information and Navigation School, Air Force Engineering University, Xi’an 710077, China; zzx20230420@outlook.com (Z.Z.); sai_liang@126.com (J.L.);

**Keywords:** polarization, deep clustering, specific emitter identification (SEI)

## Abstract

Specific Emitter Identification (SEI) distinguishes individual emitters by extracting subtle features from intercepted radio frequency signals. This process relies on the design and extraction of specific features. Current methods for selecting and characterizing radio frequency fingerprints vary by individual, and the extraction process is closely coupled with environmental conditions. As a result, the generality of such identification algorithms is often limited, particularly when the application environment does not match the premise of feature design, leading to rapid degradation or even failure of individual identification performance. This paper proposes a deep clustering model based on polarization feature learning for identifying individual communication emitters. The approach involves constructing a guided network to extract datasets of polarization features from communication signals and utilizing a contrastive representation learning network to extract dual-polarization features from I/Q data samples. Subsequently, a Bayesian nonparametric (BNP) class mixture model algorithm, capable of inferring an unknown number of clusters, is employed to build a multi-level clustering network for clustering analysis of the extracted features. Under 5 dB conditions, the method described in this paper achieves an average recognition accuracy of 87.5%.

## 1. Introduction

Currently, individual identification of communication emitters primarily employs specific emitters identification technologies [1], namely radio frequency (RF) fingerprinting technology. This technique utilizes the hardware differences of communication emitters devices, referred to as RF fingerprints [2], to distinguish and identify radiation sources based on their emitted signals in space. The technology relies on the hardware discrepancies of internal components of the communication transmitter, such as oscillators, modulators, power amplifiers, and filters, which result from the tolerance effects of the electronic components located at the front end of the transmitter’s antenna. Tolerance effects include manufacturing tolerances, which arise from deviations between the electrical parameters of electronic components and their nominal values due to factors like material and manufacturing processes, as well as drift tolerances, which are changes in electrical parameters caused by the degradation and aging of electronic components [3]. The RF fingerprints resulting from these tolerance effects are diminishing due to increasingly precise manufacturing processes and signal processing technologies, reducing the hardware differences in components like oscillators, modulators, power amplifiers, and filters within transmitters. Consequently, the information contained in such fingerprint features is becoming more similar, decreasing their distinguishability. Thus, methods focused on exploiting these fingerprint features are becoming increasingly limited in performance, making it more challenging to meet the demands of individual identification.

To address these issues, this paper investigates the hardware differences in the antennas of RF systems, specifically the polarization parameter non-idealities caused by design parameters, material composition, and manufacturing processes of different transmitting antennas, and proposes the RF fingerprint of antennas. This fingerprint is characterized through polarization features and introduced into the field of individual identification of communication emitters, thereby expanding the available feature dimensions in this field and analyzing identification methods from the perspective of new fingerprint features.

**Figure 1:**
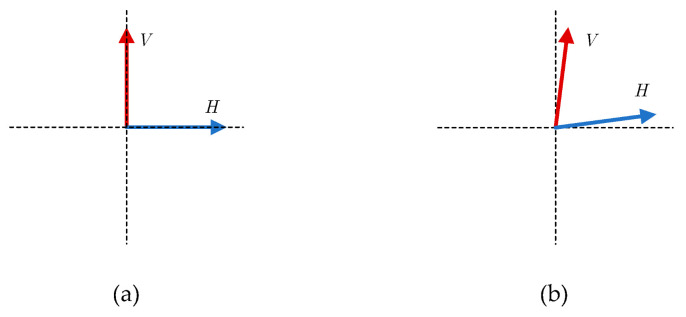
Schematic of ideal and actual antenna polarization. (**a**) Ideal antenna polarization. (**b**) Actual antenna polarization.

Polarization refers to the variation in the direction and magnitude of the electric field intensity of electromagnetic waves over time, consistent with the polarization form of the transmitting antenna [4]. The design principles of antennas dictate that the polarization state of actual antennas can never achieve the ideal state. Therefore, electromagnetic waves radiated by single-polarization antennas include, besides the desired component (main polarization component), an undesired component perpendicular to it, known as the cross-polarization component. Similarly, electromagnetic waves radiated by dual-polarization and multi-polarization antennas cannot achieve complete orthogonality in the desired polarization components. These scenarios lead to non-ideal deviations in the polarization parameters of antennas, which can be characterized as dual-polarization features described by the polarization ratio [5], i.e., the amplitude ratio and phase difference between the main polarization and cross-polarization directions. Through empirical analysis of antennas of the same manufacturer, model, and batch, subtle differences in the polarization amplitude ratio and polarization phase difference between different antennas were observed. High-performance communication systems typically require XPI (cross-polarization isolation) > 25 dB [6], reflecting the systematic deviations in antenna design and manufacturing that do not affect information transmission but are detectable and reproducible subtle feature differences. These can serve as RF fingerprint features for the individual identification of communication emitters. Figure 1 shows a schematic diagram of an ideal antenna with actual antenna polarization.

## 2. Proposed Method

The primary component of electromagnetic waves radiated by communication antennas is the main polarization component, with a small amount of orthogonally polarized components termed as cross or orthogonal polarization components. According to antenna design principles, the ratio of the power level of the main polarization component of the radiated electromagnetic waves to the total power level is referred to as the cross-polarization discrimination. This metric is crucial for evaluating system performance, where a higher cross-polarization discrimination indicates better system performance. However, in practical applications, due to inherent device errors and mechanical processing inaccuracies, antennas exhibit varying degrees of deviation in cross-polarization discrimination, which is reflected in the polarization features of communication radiation sources. The reception of polarization signals requires a polarization reconnaissance receiver, typically employing methods such as polarization-pattern diagrams, amplitude–phase methods, and multi-antenna approaches. Currently, the amplitude–phase method is widely utilized in modern receiving systems [7]. Therefore, this study utilizes the amplitude–phase method to achieve the reception of polarization signals and represents the cross-polarization discrimination of antennas through polarization ratio analysis.

### 2.1. Extraction of Dual-Polarization Features

The polarization state can be described by the shape and rotational direction of the spatial trajectory formed by the tip of the electric field vector as it varies over time. It can be categorized into three types: fully polarized waves, partially polarized waves, and unpolarized waves [8]. Any electromagnetic wave can be decomposed into a combination of a pair of orthogonal polarization components in the horizontal and vertical directions. Assuming that the electromagnetic wave emitted by a communication emitter is a monochromatic, fully polarized electromagnetic wave propagating in the positive direction along the z-axis, the vector form of the instantaneous value of the electromagnetic wave [9] is

(1) $$E=E_{V}{\vec{e}}_{V}+E_{H}{\vec{e}}_{H}$$

In the equation, $e_{V},\;e_{H}$ are the unit basis vectors in the horizontal and vertical directions, respectively.

(2) $$E_{V}=E_{V_{0}}\cos(2\pi f_{0}\cdot t-kz+\phi_{V})$$

(3) $$E_{H}=E_{H_{0}}\cos(2\pi f_{0}\cdot t-kz+\phi_{H})$$

In the equation, $f_{0}$ represents the frequency of the electromagnetic wave; K represents the spatial propagation constant; $E_{V_{0}}$ and $\phi_{V}$ represent the amplitude and phase of the electric field in the horizontal direction; $E_{H_{0}}$ and $\phi_{H}$ represent the amplitude and phase of the electric field in the vertical direction.

The complex vector form of the electromagnetic wave can be written as

(4) $$e(t,z)=e\cdot e^{j(2\pi f_{0}t-Kz)}$$

(5) $$e=\left[\begin{array}{l} e_{V}\\ e_{H} \end{array}\right]=\left[\begin{array}{l} e_{V_{0}}e^{j\phi_{V}}\\ e_{H_{0}}e^{j\phi_{H}} \end{array}\right]$$

$e_{V_{0}}$ and $\phi_{V}$ are the amplitude and phase of the electric field in the horizontal direction.

$e_{0y}$ and $\phi_{y}$ are the amplitude and phase of the electric field in the vertical direction.

From Equations (2) and (3), the polarization information is represented by the amplitude ratio and phase difference of the electric field components in the two orthogonal directions. Ignoring the absolute phase information, the polarization ratio can be defined according to the linear polarization ratio method [10] as

(6) $$\eta =\frac{e_{V}}{e_{H}}=\frac{e_{V_{0}}e^{j\phi_{y}}}{e_{H_{0}}e^{j\phi_{x}}}=\tan\gamma e^{j\phi }$$

Thus, for a given electromagnetic wave, its polarization state can be fully described by the polarization ratio. In this expression, $\tan\gamma =e_{H_{0}}/e_{V_{0}},\;\gamma \in [0,2\pi )$ represents the amplitude ratio of the vertical to horizontal electric field components. $\phi =\phi_{H}-\phi_{V},\;\phi \in [0,2\pi ]$ denotes the phase difference between the vertical and horizontal components.

The value (γ,ϕ) is the phase descriptor of the electromagnetic wave’s polarization state [11] and can be used to characterize the polarization domain features of the emitter. To more intuitively represent the polarization domain features of the communication emitter’s RF signal, the value $\tan\gamma =e_{y}/e_{x},\;\gamma \in [0,2\pi )$ is directly used as the amplitude ratio of the main polarization to the cross-polarization signal, termed the polarization amplitude ratio. The phase angle $\phi =\phi_{y}-\phi_{x},\;\phi \in [0,2\pi ]$ is used as the phase difference between the main polarization and the cross-polarization signal, termed the polarization phase difference.

Conventional communication receivers are single-polarization receivers that can only receive single-polarization signals with matched polarization. Receiving dual-polarization and multi-polarization signals requires a polarization reconnaissance receiver. Typically, polarization reconnaissance receivers employ methods such as the polarization–radiation pattern method, amplitude–phase method, and multi-antenna method, with the amplitude–phase method being widely used in modern reception systems. By calculating the amplitude and phase of the main polarization and cross-polarization signals received by the polarization reconnaissance receiver, the dual-polarization features of the communication emitter. These features are essential for distinguishing the polarization features of different emitters and enhancing the accuracy of emitter identification. The reception process for a dual-polarization receiver is as follows.

Assuming that the signal (a plane electromagnetic wave) emitted by the communication source is a continuous wave signal, as expressed in Equation (1), Equation (1) can be simplified to

(7) $$E=E_{0x}\cos(wt+\phi_{x})e_{x}+E_{0y}\cos(wt+\phi_{y})e_{y}$$

Assuming that the stable frequency of the local oscillator is $f_{1}$ and its phase is $\phi_{1}$, it can be expressed as

(8) $$U_{t}=U_{0}\cos(2\pi f_{1}\cdot t+\phi_{1})$$

The outputs of the signals from the co-polar and cross-polar channels after mixing and intermediate frequency filtering are

(9) $$E_{x1}=a\cdot E_{0x}\cos(2\pi (f_{0}-f_{1})t+\phi_{x}-\phi_{1})$$

(10) $$E_{y1}=a\cdot E_{0y}\cos(2\pi (f_{0}-f_{1})t+\phi_{y}-\phi_{1})$$

where a is the signal gain. The signals from the co-polar and cross-polar channels undergo orthogonal phase processing, producing two orthogonal I/Q signals.

The in-phase signal $U_{p}=\cos[2\pi (f_{0}-f_{1})t]$ is transformed into $U_{p}=\sin[2\pi (f_{0}-f_{1})t]$ by a 90° phase shifter.

$E_{x1},E_{y1}$ undergoes orthogonal phase processing to yield two orthogonal signals:

(11) $$E_{xI}=g\cdot a\cdot E_{0x}\cos(\phi_{x}-\phi )$$

(12) $$E_{xQ}=g\cdot a\cdot E_{0x}\sin(\phi_{x}-\phi )$$

(13) $$E_{yI}=g\cdot a\cdot E_{0y}\cos(\phi_{y}-\phi )$$

(14) $$E_{yQ}=g\cdot a\cdot E_{0y}\sin(\phi_{y}-\phi )$$

where g is the gain of the mixer and low-pass filter. The two signals undergo a transformation from A/D to $I_{n},Q_{n}(n=1,2,\cdots ,N)$.

After passing through an orthogonal dual-channel linear detector, the amplitudes of the main polarization and cross-polarization signals are obtained.

(15) $$E_{1}=g\cdot a\cdot E_{0x}$$

(16) $$E_{2}=g\cdot a\cdot E_{0y}$$

Using a single-channel coherent detector, the phase difference between the co-polarized and cross-polarized signals is obtained as

(17) $$V_{0}=g^{2}\cdot a^{2}\cdot E_{0x}\cdot E_{0y}\cos(\phi_{y}-\phi_{x})$$

Through calculation, the polarization amplitude ratio and polarization phase difference of the main polarization and cross-polarization signals are obtained as

(18) $$\chi =\frac{E_{2}}{E_{1}}=\frac{g\cdot a\cdot E_{0y}}{g\cdot a\cdot E_{0x}}=\frac{E_{0y}}{E_{0x}}$$

(19) $$\phi =\arccos(\frac{V_{0}}{E_{1}\cdot E_{2}})=\phi_{y}-\phi_{x}$$

The obtained polarization amplitude ratio and polarization phase difference represent the polarization characteristics of the individual communication radiation source, thereby enabling classification and identification.

### 2.2. Polarization Feature Learning Extraction Model

**Figure 2:**
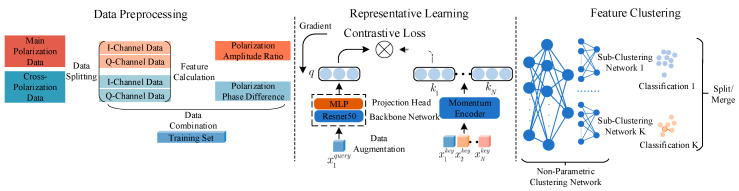
Polarization Feature Learning Extraction Model.

The communication emitter classification model designed in this paper, based on feature learning and extraction in the polarization domain, is divided into three modules: the data preprocessing module, the representation learning module, and the feature clustering module, as shown in Figure 2.

### 2.3. Data Preprocessing

The polarization reconnaissance receiver captures polarized signals containing two sets of I/Q data for the main polarization and cross-polarization channels. Directly processing these four-channel I/Q data using a deep clustering network can make the network susceptible to noise and errors, hindering its ability to quickly capture polarization features in the signal. To enable the deep clustering network to extract polarization features quickly and stably, this paper constructs training and testing datasets that guide the network to adapt to polarization features through preprocessing. The preprocessing is divided into three steps. First, the received main and cross-polarization signals’ RF I/Q complex data are split into two sets of I and Q real-valued data. Then, using the corresponding sets of I and Q data, the amplitude and phase of the main and cross-polarization signals are calculated. Next, the polarization amplitude ratio and polarization phase difference between the main and cross-polarization signals are computed using the two sets of amplitude and phase values. Finally, the polarization amplitude ratio and polarization phase difference from the main and cross-polarization signals are aligned with the original four-channel I/Q data, concatenated and segmented, resulting in an M × 6 × 128 dimensional training and testing dataset.

### 2.4. Deep Clustering Network

**Figure 3:**
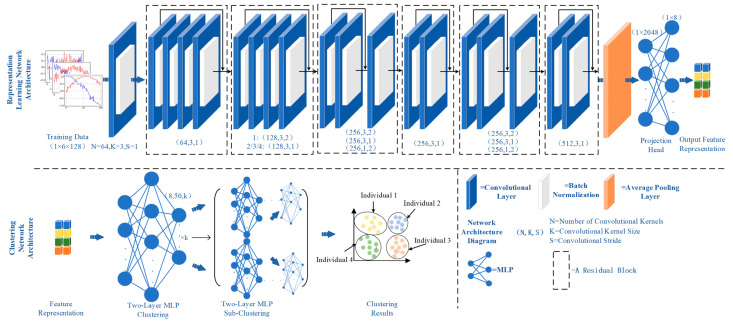
Structure of the Deep Clustering Network Based on Representation Learning.

This paper designs a deep clustering network based on contrastive representation learning, which is divided into two stages: the representation learning stage and the feature clustering stage. In the Algorithm 1, a contrastive learning model [12,13,14] is adopted, consisting of four parts: data augmentation, a basic encoding network, a projection head, and a contrastive loss. The data augmentation strategies include random cropping with row and column flipping and length adjustments, along with central value smoothing, enhancing the model’s generalizability and robustness and improving the effectiveness of feature extraction from the raw data. The basic encoding network utilizes Momentum Contrast (MoCo) [15,16] comprising a sequence and a momentum encoder. The sequence ensures that the training data from the current mini-batch enters the sequence and pushes out the earliest batch data, achieving a stack-like processing. The momentum encoder establishes a connection between the key encoders at time t and t-1, ensuring all key encoders acquire a similar structure. These structures encode the augmented data, mapping it to a representation in the latent space. The projection head, a multilayer perceptron, learns more complex nonlinear mappings than traditional fully connected networks, thereby better capturing the relationships between features. Further encoding by the projection head enhances the distinctiveness of the representations in the latent space, improving contrastive performance. The contrastive loss uses the classical contrastive learning loss function Noise Contrastive Estimation (InfoNCE) [17], aiming to maximize consistency between two related features, bringing representations of positive samples closer and driving those of negative samples further apart, effectively training the basic encoding network and projection head. The network structure is shown in Figure 3.
**Algorithm 1: Representation learning algorithm process.**Step 1: Input: Dataset X, sequence q, basic encoding networks f_q and f_k, momentum m. Step 2: Initialize the network: The parameters and weights of networks f_q and f_k are kept consistent. Step 3: For x in X do: Step 4:     Extract data augmented using a randomly selected augmentation method: x_q = aug(x) Step 5:     Select data augmented using other enhancement methods: x_k = aug(x) Step 6:     The q encoder encodes to obtain the query feature set: q = f_q(x_q) Step 7:     The k encoder encodes to obtain the feature dictionary: k = f_k(x_k) Step 8:     Input the augmented data features into the feature dictionary one by one: k += k Step 9:     Positive sample similarity calculation: l_p = f(q(·), k + (·)) Step 10:    Negative sample similarity calculation: l_n = f(q(·), k − (·)) Step 11:    similarity merging: l = [l_p,l_n] Step 12:    Set the sample label to 0: labels = zeros(N) Step 13:    Calculate the InfoNCE loss function and update the basic encoding network through backpropagation f_q Step 14:    Momentum encoder update: f_k = m×f_k + (1 − m)×f_q Step 15:    Update the feature dictionary: Enter the sequence(q, k); Exit the sequence(q) Step 16: End for Step 17: Output: Features extracted through representation learning.

### 2.5. Network Update Optimization

During the feature clustering stage, namely Algorithm 2, given the features of high-dimensional discrete data from polarized signals and the ability of deep neural networks to represent high-dimensional data, an adaptive deep nonparametric clustering method suitable for an unknown number of clusters was employed. This method is a type of Dirichlet Process Mixture (DPM) [18] algorithm, which creates subclusters for each cluster and modifies the number of clusters through splitting and merging. When splitting a cluster, each cluster is probabilistically divided into two subclusters with a probability P1. This division adapts to changes in the variable k by duplicating the k-th unit in the last layer of the clustering network and the weights connected to the previous hidden layer. The parameters learned from the subcluster network are then used to initialize the two new clusters. The expression for the above process is as follows:

(20) $$P_{1}=\mathrm{Min}(1,H_{s})$$

(21) $$H_{s}=\frac{\alpha \Gamma (N_{k,1})f_{x}(\chi_{k,1};\lambda )\Gamma (N_{k,2})f_{x}(\chi_{k,2};\lambda )}{\Gamma (N_{k})f_{x}(\chi_{k};\lambda )}$$

**Algorithm 2: Clustering algorithm process.**Step 1: Input: Dataset S, clustering C, prerequisite tasks τ, deep neural networks $\Phi_{\theta }$ and $\Phi_{\varphi }$.
Step 2: Train the network: Use the prerequisite task τ to optimize and update the deep neural networks.         »The prerequisite task is feature extraction during the representation learning stage (Equation (27)). Step 3: For $x_{i}\in S$ do
Step 4:    $N_{s}\cup N_{x_{i}}\to N_{s}$, in this context $N_{x_{i}}=\Phi_{\theta }(x_{i})$ are the k nearest neighbors of $N_{s}$             »$N_{s}$ is the set of nearest neighbors Step 5: End for
Step 6: while $Loss↓$ do       »KL Divergence optimization for the clustering network (Equations (28) and (29))
Step 7:    Update the deep neural network $\Phi_{\varphi }$ Step 8: End while
Step 9: while $Len(Y)↑$ do       »Isotropic loss optimization for the subcluster network (Equations (30)–(32))
Step 10:    $(\Phi_{\varphi }(S)>\eta )\to Y$
Step 11:    Update the deep neural network $\Phi_{\varphi }$ Step 12: End while
Step 13: Renturn: $\Phi_{\varphi }(S)$       »Divide the dataset S into corresponding clustering clusters C


In this context, the Hastings ratio $H_{s}$ is calculated as the ratio of the marginal likelihoods of the data under two subclusters to the marginal likelihood of the data under the original cluster. Γ represents the gamma function. $N_{k,j}$ represents the data in cluster $j(j\in \left\{1,2\right\})$, in this context $N_{k,j}=\left|\chi_{k,j}\right|$, $\chi_{k,j}={\left(x_{i}\right)}_{i:(z_{i},{\tilde{z}}_{i})=(k,j)}$. $f_{x}(•,\lambda )$ is the marginal likelihood function. λ=(m,κ,ψ,v) represents the normal-inverse-Wishart (NIW) hyperparameters. The purpose of the splitting operation is to decompose a cluster into two finer sub-clusters; it is triggered when the data distribution within that cluster exhibits multimodal characteristics. Every 10 training iterations, the algorithm evaluates the likelihood of splitting for each existing cluster. For a cluster, the Hastings ratio for its split is calculated, where the probability of splitting is denoted by $P_{1}$. The numerator is the marginal likelihood of the data under the assumption that it is split into two sub-clusters, and the denominator is the marginal likelihood of the data under the original single cluster. When the Hastings ratio is greater than or equal to 1, the network is split; when it is less than 1, the decision to split is made randomly. When it is decided to split cluster k, the algorithm duplicates the kth unit in the final layer of the clustering network and copies its weights connecting to the previous hidden layer. These two new units are initialized as the output nodes of the two sub-clusters, respectively. Following the split, the number of clusters k is increased by 1. The two newly created sub-clusters learn their initial parameters through the sub-clustering network. The parameters are initialized randomly. Following the split, the original cluster center is replaced by two new cluster centers.

During the merging of clusters, to prevent the simultaneous merging of two subclusters which could lead to multi-class aggregation errors, and to maximize the efficiency of subcluster merging, each merging operation proceeds with a probability $P_{2}$ of merging each subcluster with its three nearest neighbors. After merging, a new subcluster network is initialized. The units from the last layer of the merged clusters, along with their weights connecting to the previous hidden layer, are removed from the clustering network. The parameters and weights of the newly formed cluster are initialized using a weighted maximum a posteriori (MAP) estimation. The weighting is based on the outputs of the deep network. The expression for the above process is as follows:

(22) $$P_{2}=H_{m}=\frac{1}{H_{s}}$$

(23) $$\kappa_{k}^{*}=\kappa +\sum_{i=1}^{N}r_{i,k}$$

(24) $$m_{k}^{*}=\frac{1}{\kappa_{k}^{*}}\left[\kappa m+\sum_{i=1}^{N}r_{i,k}x_{i}\right]$$

(25) $$v_{k}^{*}=v+\sum_{i=1}^{N}r_{i,k}$$

(26) $$\psi_{k}^{*}=\frac{1}{v^{*}}\left[v\psi +\kappa mm^{T}+(\sum_{i=1}^{N}r_{i,k}x_{i}x_{i}^{T})-\kappa_{k}^{*}m_{k}^{*}{\left(m_{k}^{*}\right)}^{T}\right]$$

The purpose of the merging operation is to combine two clusters with high similarity into a single cluster, thereby avoiding fragmentation caused by overly fine-grained partitioning. Every 10 training iterations, the algorithm identifies the three nearest neighbors for each cluster (based on the Euclidean distance between cluster centers). For each candidate pair, the Hastings ratio for merging is calculated, and the probability of merging is denoted by b. That is, merging occurs whenever it improves the overall likelihood; otherwise, the decision is made randomly with probability $P_{2}$.

The network consists of two main parts. The first part is a clustering network, and the second part consists of K subcluster networks, where K represents the number of clusters. These networks are trained through the Expectation–Maximization (EM) [19] process used in mixture models.

The update optimization of the deep clustering network based on contrastive representation learning is divided into two steps. The first step is the network update optimization during the representation learning phase, using the InfoNCE loss function, which is expressed as follows:

(27) $$L_{InfoNCE}=-\log\frac{\exp(f(q,k^{+})/\tau )}{\exp(f(q,k^{+})/\tau )+\sum_{i=1}^{N-1}\exp(f(q,k_{i}^{-})/\tau )}$$

where q represents the embedding of the query sample, $k^{+}$ represents the embedding of the positive sample, $k_{i}^{-}$ represents the embedding of the negative sample, τ is the temperature hyperparameter, and dot product f(•) is used for similarity calculation. In the contrastive learning architecture described in the paper, the backbone network utilizes the standard ResNet-18 network. The polarization characteristics of radiation sources are typically subtle and embedded within complex signal structures. ResNet-18 was selected because its residual connection architecture can effectively extract deep-layer features while mitigating the vanishing gradient problem during training. This architecture has been widely adopted in signal processing tasks involving high-dimensional inputs and has demonstrated strong performance in relevant studies. A two-layer MLP network is added as the projection head on top of the backbone, with the first layer having 2048 neurons and the second layer having an output feature dimension of 8, using the ReLU function for activation in the output layer. A multi-layer perceptron is employed to project features into a latent space suitable for clustering. The choice of a two-layer structure follows established best practices in the literature, striking a balance between model complexity and generalization ability while avoiding overfitting on the given dataset. Other hyperparameters of the network include a batch size of 128 and a temperature hyperparameter τ=0.2. We adopted hyperparameter settings that have been validated in similar tasks, ensuring that these values align with the task characteristics and are consistent with existing practices. This approach ensures stable training of both the ResNet and MLP components.

The second step is the network update optimization during the feature clustering stage, which employs two optimization methods. The first method involves KL divergence targeting the E-step of the EM process in the mixture model, and its expression is as follows:

(28) $$L_{c1}=\sum_{i=1}^{N}KL(\left.r_{i}\right\|r_{i}^{E})$$

(29) $$r_{i}={\left(r_{i,k}\right)}_{k=1}^{K}$$

where $r_{i,k}$ represents the soft assignments of feature representations, i.e., the mapping from feature representations to clusters.

The second method is isotropic loss targeting the M-step of the EM process in the mixture model, and its expression is as follows:

(30) $$L_{sub}=\sum_{k=1}^{K}\sum_{i=1}^{N_{k}}\sum_{j=1}^{2}\left.{\tilde{r}}_{i,j}\right\|x_{i}-{\left.{\tilde{\mu }}_{k,j}\right\|}_{l_{2}}^{2}$$

(31) $$N_{k}=\left|\chi_{k}\right|=\left|{\left(x_{i}\right)}_{i:z_{i}=k}\right|$$

(32) $$z_{i}=\arg\max_{k}(r_{i,k})$$

where $x_{i}$ represents the feature representation of a sample, and ${\tilde{\mu }}_{k,j}$ is the mean of subcluster s in cluster c. In the clustering architecture described in the paper, the MLP consists of an input layer, a single hidden layer, and an output layer. The input layer has 8 neurons (the input data dimension for the clustering module), the hidden layer has 50 neurons (which can be set to a different number with minimal impact on clustering results), and the number of neurons in the output layer is k, which changes with the clustering results.

Other hyperparameters of the network are set as follows: batchsize = 128, learning rate for the clustering network lr = 0.0005, and learning rate for the subcluster network lr = 0.005.

In network performance evaluation, three metrics are used: Accuracy (ACC), Normalized Mutual Information (NMI), and Adjusted Rand Index (ARI). Accuracy (ACC) refers to the ratio of samples whose true labels correctly match the predicted clustering labels. The formula for calculating accuracy is as follows:

(33) $$ACC=\underset{m}{\max}(\frac{\sum_{i=1}^{N}\left(y_{i}=l(m(z_{i}))\right)}{N})$$

where N is the number of samples, $y_{i}$ is the true label of the sample, $z_{i}$ is the label assigned by the clustering algorithm, l(•) is the indicator function, and m is the number of matches between the clustering labels assigned by the algorithm and the true labels.

Normalized Mutual Information (NMI) measures the mutual information between the predicted clustering labels and the true labels of the samples, normalized by the entropy of these labels. The formula for calculating NMI is as follows:

(34) $$NMI=\frac{2\times I(y,z)}{H(y)+H(z)}$$

In this context, $z={\left(z_{i}\right)}_{i=1}^{N}$, $y={\left(y_{i}\right)}_{i=1}^{N}$. H(•) is the entropy function, and I(•) is the mutual information function.

The Adjusted Rand Index (ARI) measures the percentage of correct assignments made for each pair of samples. The formula for calculating ARI is as follows:

(35) $$ARI=\frac{RI-E(RI)}{\max(RI)-E(RI)}$$

(36) $$RI=\frac{TP+TN}{TP+FP+TN+FN}$$

where TP is the number of correct decisions to place similar samples in the same category. TN is the number of correct decisions to place dissimilar samples in different categories. FP is the number of incorrect decisions to place dissimilar samples in the same category, and FN is the number of incorrect decisions to place similar samples in different categories.

## 3. Experimental Results and Discussion

### 3.1. Construct Real Experimental Scenarios

**Figure 4:**
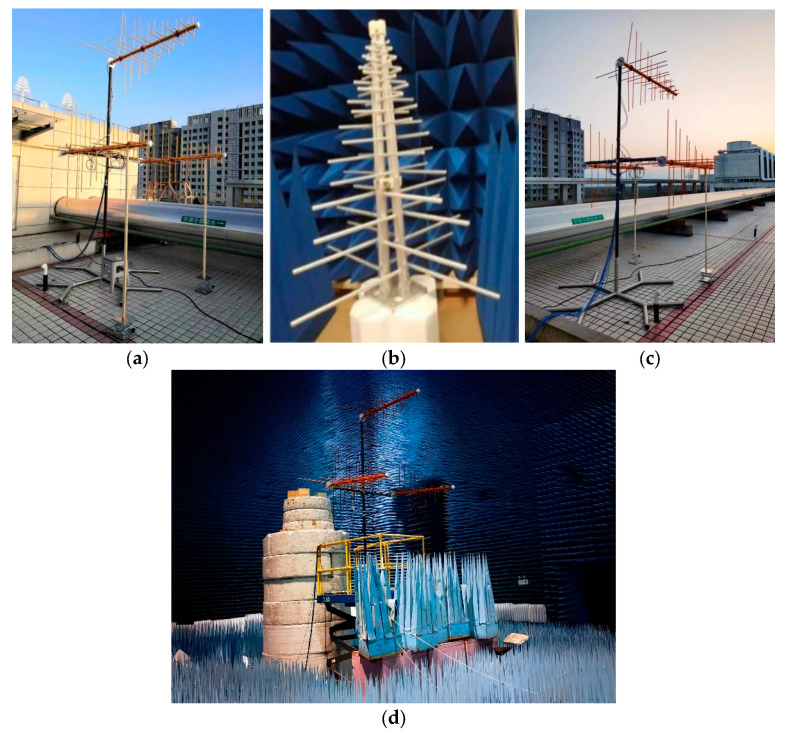
Receiving antenna structure: (**a**) dual-polarized log-periodic antenna; (**b**) outdoor build the antenna right behind; (**c**) outdoor set up antenna left front; (**d**) anechoic chamber antenna to build antennas. The text in the figure is irrelevant to this article.

In order to verify the effect of the proposed method in practical engineering application, a communication radiation source identification test system was built, which consists of three parts, the baseband signal generation part, the RF signal transmission part and the RF signal acquisition and processing part. The baseband signal generation part is mainly composed of a dual-band signal generator, which supports multi-channel, multi-style modulated baseband signal generation, RF modulation, broadband frequency conversion and RF output of baseband signal; The radio frequency signal transmission part is mainly composed of four pairs of a knife antenna (vertical polarization) of the same manufacturer, the same model and the same batch, which can complete the space radiation of the radio frequency signal, and the electromagnetic wave radiated is vertically polarized; The RF signal acquisition and processing part is mainly composed of a data acquisition platform, which has the functions of receiving electromagnetic wave signals in the bandwidth range of 100 MHz~400 MHz. The primary objective of this study is to validate the feasibility of the proposed polarization-feature-based clustering algorithm under controlled conditions. By using antennas from the same manufacturer and model, as shown in Figure 4, we have deliberately minimized hardware variations so as to evaluate the algorithm’s performance in polarization feature extraction and clustering without introducing confounding factors. It should be noted that during the generation and acquisition of the experimental data in this paper, no adjustments were made to account for differences in the time-domain characteristics of individual radiation sources; all sources maintained consistent time-domain waveform structures, meaning the experiments were conducted under conditions where time-frequency characteristics were difficult to distinguish. This study focuses on verifying the feasibility of using polarization characteristics for radiation source identification. This study aims to demonstrate the feasibility of polarization characteristics under ideal conditions, without taking into account real-world channel complications such as multipath effects, receiver motion, polarization mismatch and frequency drift.

The experimental parameter settings are as follows: the symbol rate of the generated signal is set to 10 Kbps, with a roll-off factor of 0.1, and the modulation style is BPSK. The carrier frequency is set to 250 MHz. The transmission uses four identical scimitar-shaped antennas from the same manufacturer, model, and production batch. The data collection platform has a sampling rate of 400 Mbit/s and a sampling duration of 2.5 ms. Each antenna collects a data volume of 100 Kbit from the radiated signals. The collected data from all four antennas are then subjected to mixing, filtering, and adding noise to produce real experimental radiation source data with a signal-to-noise ratio ranging from −5 dB to 20 dB in Experiment 1. In Experiment 2, the modulation style of the generated signals is changed to BPSK, 2ASK, FM, and AM using the same four scimitar-shaped antennas. Data is collected in the same amount as in Experiment 1 and undergoes similar processing of mixing, filtering, and adding noise to produce real experimental variable-modulation data. The statistical data and experimental results are all averages of 10 independent runs, with a different random seed used for each run.

### 3.2. Experiment 1: Verification of Individual Identification Performance Under Different Signal-to-Noise Ratios

**Figure 5:**
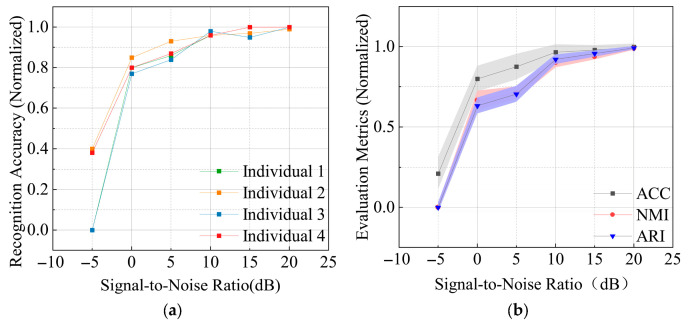
Classification performance of actual collected data at different signal-to-noise ratios: (**a**) classification accuracy; (**b**) classification evaluation metrics.

The classification accuracy and the optimized evaluation metrics of actual collected data under different signal-to-noise ratios are shown in Figure 5a,b, respectively. From the figures, it can be observed that for actual collected data at a signal-to-noise ratio of 0 dB, the individual identification accuracy reaches 75%, with an average recognition rate exceeding 80%. As the signal-to-noise ratio increases, the individual identification accuracy gradually approaches 100%, and the three evaluation metrics of this method also gradually approach 1. This demonstrates that the method achieves robust identification performance with real-data.

**Figure 6:**
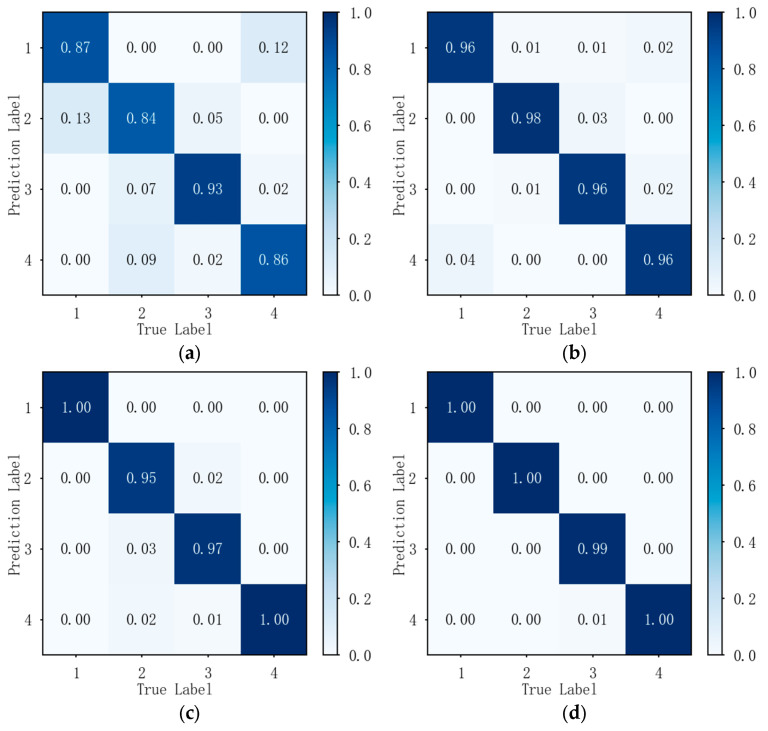
Classification confusion matrix for actual collected data at different signal-to-noise ratios: (**a**) confusion matrix at 5 dB signal-to-noise ratio; (**b**) confusion matrix at 10 dB signal-to-noise ratio; (**c**) confusion matrix at 15 dB signal-to-noise ratio; (**d**) confusion matrix at 20 dB signal-to-noise ratio.

Figure 6 presents the confusion matrices for individual classification and recognition of real-world data at signal-to-noise ratios ranging from 5 dB to 20 dB. Through the comparison of four sub-figures, it can be concluded that when the signal-to-noise ratio of the real data exceeds 5 dB, this method can effectively distinguish between four radiation source individuals, and the classification performance improves as the signal-to-noise ratio increases. Under 5 dB conditions, the method described in this paper achieves an average recognition accuracy of 87.5%, with individual recognition accuracy ranging from a minimum of 84% to a maximum of 93%.

**Figure 7:**
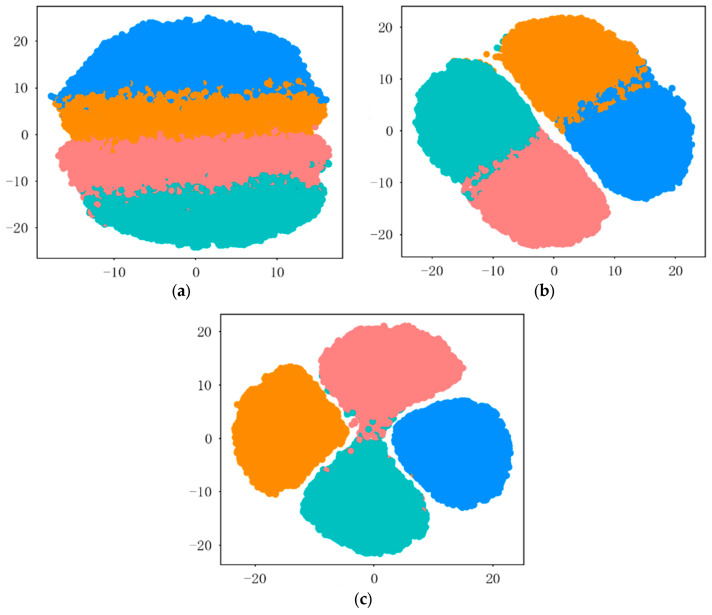
Dimensionality reduction distribution of feature clustering results at different signal-to-noise ratios using real data: (**a**) dimensionality reduction distribution of clustering features at 5 dB signal-to-noise ratio; (**b**) dimensionality reduction distribution of clustering features at 10 dB signal-to-noise ratio; (**c**) dimensionality reduction distribution of clustering features at 15 dB signal-to-noise ratio.

The dimensionality-reduced distributions of feature clusters extracted by this method from real data at various signal-to-noise ratios are shown in Figure 7a–c. The figures demonstrate that as the signal-to-noise ratio increases, the distances between the feature distributions of the four radiation source individuals gradually widen, and the boundaries become clearer. This proves that the features extracted by this method have high distinguishability, meeting the requirements for individual identification.

### 3.3. Experiment 2: Verification of Individual Identification Performance Under Varying Modulation Styles

**Figure 8:**
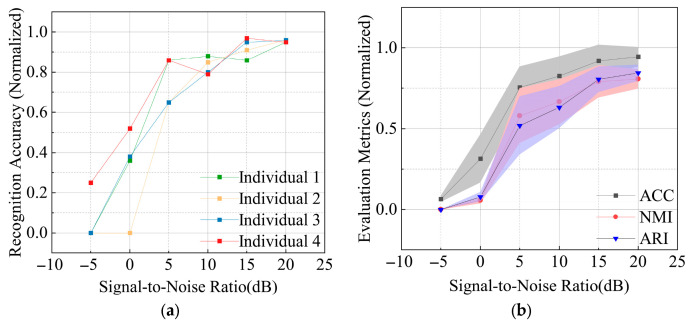
Performance of classification and recognition for real-world modulated individuals under different signal-to-noise ratios: (**a**) classification accuracy under different signal-to-noise ratios; (**b**) classification evaluation metrics under different signal-to-noise ratios.

The classification accuracy and evaluation metrics for individual identification of radiation sources with varying modulation styles, using real-world data, are shown respectively in Figure 8a,b. From the figures, it can be seen that the four modulated radiation source individuals achieve an average recognition rate of over 75% at a signal-to-noise ratio of 5 dB. As the signal-to-noise ratio increases, both the recognition accuracy and the optimized evaluation metrics of this method improve gradually. This demonstrates the method’s good adaptability in identifying individuals with variable modulation styles.

**Figure 9:**
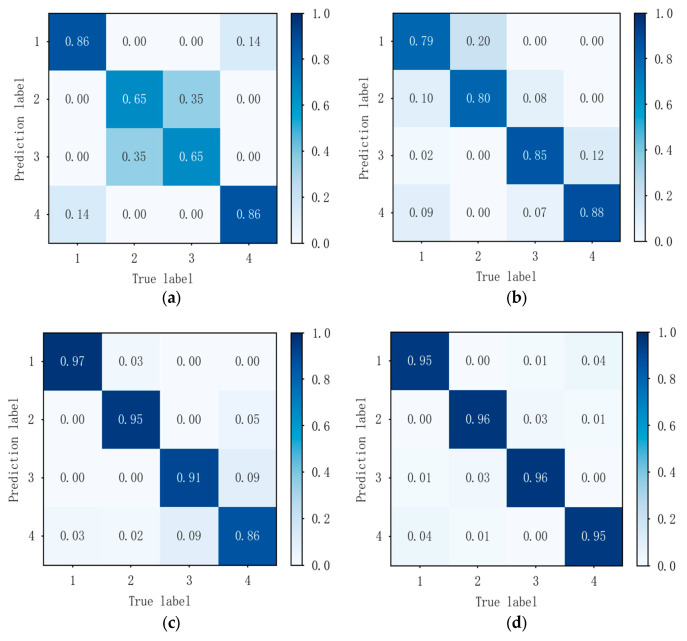
Classification confusion matrix for real modulated data at different signal-to-noise ratios: (**a**) confusion matrix at 5 dB signal-to-noise ratio; (**b**) confusion matrix at 10 dB signal-to-noise ratio; (**c**) confusion matrix at 15 dB signal-to-noise ratio; (**d**) confusion matrix at 20 dB signal-to-noise ratio.

Figure 9 presents the confusion matrices for individual classification and recognition of real-world modulated data at signal-to-noise ratios ranging from 5 dB to 20 dB. By comparing the four sub-figures, it is evident that for radiation sources with varied modulation styles, this method can distinguish between the four sources when the signal-to-noise ratio exceeds 5 dB, and the distinction improves as the signal-to-noise ratio increases.

**Figure 10:**
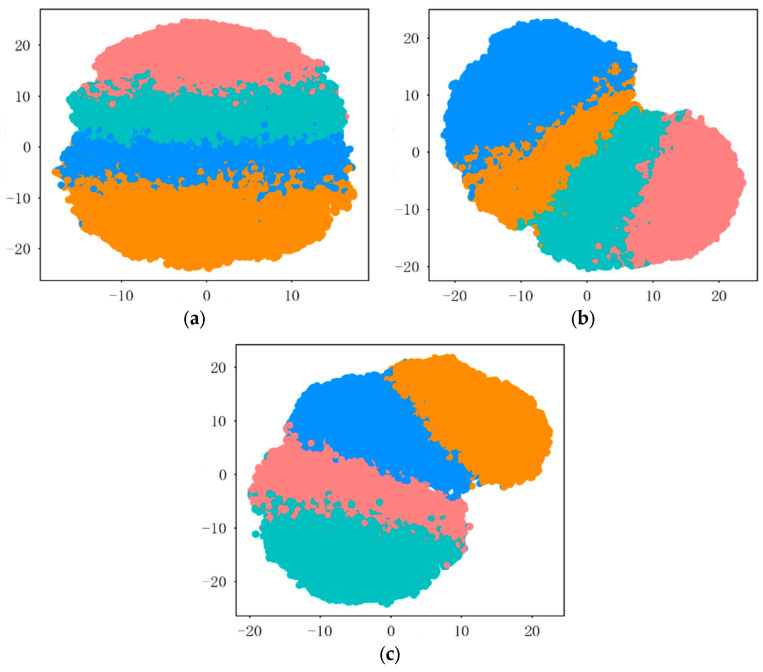
Dimensionality reduction distribution of feature clustering results for real-world modulated data at different signal-to-noise ratios: (**a**) dimensionality reduction distribution of clustering features at 5 dB signal-to-noise ratio; (**b**) dimensionality reduction distribution of clustering features at 10 dB signal-to-noise ratio; (**c**) dimensionality reduction distribution of clustering features at 15 dB signal-to-noise ratio.

Figure 10 shows the dimensionality reduction distribution of feature clustering results for real-world modulated data at different signal-to-noise ratios. Comparing the three sub-figures, it can be observed that as the signal-to-noise ratio increases, the feature distributions of the four radiation sources gradually converge, and the overlapping areas decrease. This demonstrates that the features extracted by this method also possess good distinguishability for radiation sources with varied modulation styles.

## 4. Conclusions

Considering the problem that individuals with less differentiated features in the spatial, temporal, frequency, and energy domains are less effective in using current communication radiation source individual identification methods, this paper proposes a communication radiation source classification method based on polarization domain feature learning extraction. On the basis of traditional individual identification of communication radiation sources using polarization domain features, the method introduces a deep clustering network based on representation learning, and obtains the polarization features in the original I/Q signal through the powerful feature extraction capability of representation learning, and then utilizes a kind of Bayesian nonparametric, BNP, or BNP, which is adaptive to the data and can be inferred mathematically for an unknown number of clusters. nonparametric (BNP) clustering method for unknown number of clusters, self-adaptive data and mathematically reasonable Bayesian nonparametric (BNP) clustering method, through the multilevel clustering network as well as split and merge operations, to achieve the autonomous clustering analysis of the polarization features of the individual communication radiation source, and complete the autonomous classification of the individual communication radiation source. Experiments have shown that the method described in this paper achieves an average classification accuracy of 87.5% for simulated radiation sources at a signal-to-noise ratio of 5 dB. It also performs well for communication radiation sources with different modulation schemes. The method described in this paper addresses the problem of classifying and identifying individual communication transmitters under conditions of low signal-to-noise ratio and variable modulation schemes. However, because the number of clusters must be determined independently, the time complexity is relatively high. Furthermore, the data used which consists solely of four antennas from the same manufacturer and model has certain limitations. In the next step, we will simplify the network structure to improve computational speed and conduct further research. In subsequent applications, practical implementation can be achieved through methods such as offline training or mini-batch training.

## Data Availability

The data in this article is classified and therefore not public.
